# Serum albumin-carcinoembryonic antigen ratio as an effective clinical tool for predicting recurrence and overall survival in patients with rectal cancer

**DOI:** 10.3389/fnut.2024.1521691

**Published:** 2025-01-17

**Authors:** Hailun Xie, Lishuang Wei, Shuangyi Tang, Jialiang Gan

**Affiliations:** ^1^Department of Gastrointestinal and Gland Surgery, The First Affiliated Hospital, Guangxi Medical University, Nanning, Guangxi, China; ^2^Guangxi Key Laboratory of Enhanced Recovery After Surgery for Gastrointestinal Cancer, Nanning, Guangxi, China; ^3^Department of Geriatric Respiratory Disease Ward, The First Affiliated Hospital, Guangxi Medical University, Nanning, Guangxi, China; ^4^Department of Pharmacy, The First Affiliated Hospital, Guangxi Medical University, Nanning, Guangxi, China; ^5^Department of Colorectal and Anal Surgery, The First Affiliated Hospital, Guangxi Medical University, Nanning, Guangxi, China

**Keywords:** albumin, carcinoembryonic antigen, nutrition, rectal cancer, recurrence, overall survival

## Abstract

**Background:**

The albumin–carcinoembryonic antigen ratio (ACR), leveraging the strengths of albumin and CEA, has emerged as a promising serum prognostic marker. However, no studies to date have explored the association between ACR and the prognosis of patients with rectal cancer. This study aimed to determine the value of albumin–carcinoembryonic antigen ratio (ACR) in predicting the progression-free survival (PFS) and overall survival (OS) of patients with rectal cancer.

**Methods:**

Survival analysis was conducted using the Kaplan–Meier method, and hazard ratios (HR) were calculated using Cox regression analyses. Nomograms were created based on variables with *p* < 0.05 in the multivariate Cox regression analysis. The predictive ability of the model was evaluated using the C-index and calibration curve, and its prognostic predictive abilities were compared to those of traditional Tumor Node Metastasis (TNM) stage using discriminant indices.

**Results:**

A total of 736 patients with rectal cancer were included in the study. ACR was significantly higher in patients with poor survival or cancer recurrence. A low ACR was associated with increased tumor invasiveness, longer hospital stays, and higher hospitalization costs. Patients with a high ACR had significantly better PFS (62.9% vs. 35.2%, *p* < 0.001) and OS (67.0% vs. 37.2%, p < 0.001) than those with a low ACR. ACR can serve as an effective auxiliary tool for pathological staging, especially in patients with stage III–IV disease. The relationship between ACR and mortality risk was L-shaped. ACR is an independent prognostic factor for PFS [HR = 0.581, 95% confidence interval (CI): 0.458–0.738, *p* < 0.001] and OS (HR = 0.560, 95% CI: 0.435–0.720, *p* < 0.001) in rectal cancer patients. ACR-based nomograms have good predictive accuracy and outperform traditional TNM stage in predicting prognosis.

**Conclusion:**

Albumin–carcinoembryonic antigen ratio is a simple and effective clinical tool for predicting the recurrence and survival of patients with rectal cancer and is a useful supplement to the TNM stage.

## Background

Rectal cancer is one of the most common malignant tumors of the gastrointestinal tract, posing a serious threat to public health and a significant burden on families and society. In 2020, approximately two million new cases and one million deaths from colorectal cancer (CRC) were reported worldwide, making it the third most common cancer and the second most common cause of cancer-related mortality (1, 2). The rectal cancer accounts for approximately 37% of CRC cases. In China, the incidence and mortality rate of rectal cancer are gradually increasing due to changes in dietary structure and aging populations. Early detection and treatment can significantly improve the prognosis of rectal cancer patients. Although new treatments and drugs for cancer have been developed over the last decade, the overall survival (OS) remains unsatisfactory. Therefore, identifying the prognostic factors for disease progression and survival is of great value and can help clinicians develop the best treatment plan for patients with rectal cancer.

The prognosis of rectal cancer is closely related to tumor-related factors such as pathological staging and specific histological and molecular features (3–5). However, owing to the heterogeneity of tumors, there are significant differences in outcomes and treatment responses, even among patients in the same stage. Therefore, the identification of simple and effective prognostic markers to help identify high-risk patients with poor prognosis has enormous clinical value. In recent years, easily accessible and non-invasive blood biomarkers have attracted increasing attention for predicting the prognosis of cancer and guiding treatment. Many prognostic indicators based on peripheral blood parameters have been developed and proven to be useful predictors of prognosis in cancer patients (6–8). A novel indicator, albumin–carcinoembryonic antigen ratio (ACR), has been developed and reported to be closely correlated to the prognosis of CRC patients. ACR comprises two parameters: albumin and CEA. Albumin is one of the most commonly used indicators for assessing a patient’s nutritional status and has a wide range of potential applications in predicting the prognosis of cancer patients. Several studies have confirmed that albumin can effectively predict the prognosis of many malignant tumors (9–11). Serum albumin levels are closely associated with systemic inflammation (12). As systemic inflammatory responses are one of the main causes of tumor development, they are critical for the prognosis of cancer patients (8, 13, 14). CEA is the most commonly used tumor marker for the diagnosis, monitoring, and prognostic prediction of CRC patients (15, 16). However, it lacks specificity in these patients. Approximately one-third of patients exhibit elevated CEA levels at the time of diagnosis, which significantly compromises its clinical application (17). ACR, which combines the advantages of albumin and CEA, has emerged as a serum prognostic marker with broad prospects. This is because albumin reflects the nutritional and inflammatory status of the host, whereas CEA reflects the tumor load.

The value of ACR as a prognostic marker for malignant tumors is still under investigation. To the best of our knowledge, no studies have reported a relationship between ACR and the prognosis of rectal cancer patients. Therefore, further research is required to fully understand their application in this population. This single-center retrospective study aimed to evaluate the value of ACR in predicting the progression-free survival (PFS) and OS of rectal cancer patients.

## Materials and methods

### Study population

This study retrospectively investigated rectal cancer patients treated at the First Affiliated Hospital of Guangxi Medical University between 2012 and 2015. Patients were selected based on the following inclusion criteria: (1) patients with complete peripheral blood cell count and serum CEA data; (2) patients aged 18 years or older; and (3) patients with confirmed diagnosis of rectal cancer by histology or cytology. Patients with other types of malignant tumors; patients who received preoperative radiation or chemotherapy; patients with autoimmune diseases, systemic infections, or inflammation; patients who took anti-inflammatory drugs within 1 week before surgery; critically ill patients with heart failure, renal failure, or other severe conditions; and patients with incomplete follow-up data were excluded from the analysis. The study was conducted in accordance with the principles of the Declaration of Helsinki. The study was approved by the Ethics Committee of the First Affiliated Hospital of Guangxi Medical University. All eligible patients and their family members signed a written informed consent form. Prior to the analysis, patient records were anonymized and de-identified.

### Clinical parameters and laboratory results

Baseline clinical variables, including sex, age, height, weight, comorbidities (hypertension and diabetes), and postoperative radiotherapy and chemotherapy, were collected. Diabetes was defined based on self-reported diabetes history, use of diabetes medication, or a fasting blood glucose of ≥126 mg/dL; hypertension was defined based on previous hypertension diagnosis and/or current use of antihypertensive medication. Tumor pathology information includes pT staging, pN staging, Tumor Node Metastasis (TNM) stage (American Joint Committee on Cancer, version 8), tumor size (diameter < 5 cm, ≥5 cm), nerve/vascular invasion, pathology type, and differentiation. Laboratory data, including complete blood count, albumin level, and tumor marker level, were obtained by peripheral venous puncture and blood sampling within 1 week before surgery. ACR was defined as the ratio of albumin (g/L) and tumor marker (CEA, mg/L) levels.

### Follow-up and outcome

Survival status (alive/dead) and recurrence (yes/no) were documented by reviewing outpatient clinical records or directly contacting patients or their relatives via phone. Follow-up visits were scheduled every 3 months during the first 2 years after surgery and every 6 months thereafter, with the last follow-up conducted in July 2021. Relapse-free survival is defined as the time interval between curative surgery and first recurrence, death, or the last follow-up. OS is defined as the time interval between curative surgery and death or the last follow-up.

### Statistical analysis

The chi-square test was used to compare categorical variables, which are presented as frequencies and proportions. The Student’s t-test was used to analyze continuous variables, which are presented as means and standard deviations. We used a restricted cubic spline function to assess the effect of ACR as a continuous variable for survival. A receiver operating characteristic (ROC) curve with Youden index was calculated to identify the optimal cut-off values for analyzing OS (maximum sensitivity and specificity). We conducted survival analysis using the Kaplan–Meier method and compared survival differences using the log-rank test. Univariate and multivariate Cox regression analyses were performed to calculate the hazard ratios (HRs) and corresponding 95% confidence intervals (CIs). Variables with *p* < 0.05 in the multivariate Cox regression analysis were incorporated into the prediction model for predicting the 1–5-year outcome of patients with rectal cancer. The predictive ability of the model was evaluated using the C-index and calibration curve. The discriminant index, consisting of the C-statistic, continuous net reclassification improvement (cNRI), integrated discrimination improvement (IDI), and time-dependent ROC curve, was used to compare the prognostic predictive abilities of the prediction model and traditional TNM stage. Statistical significance was defined as a two-sided *p*-value of <0.05. Statistical analysis was conducted using SPSS software (version 24.0).

## Results

### Demographic characteristics

This study included 736 patients diagnosed with rectal cancer, 461 of whom were male (62.6%) and 275 were female (37.4%). Their mean age was 58.15 ± 12.87 years. TNM stage revealed that 171, 231, 285, and 49 patients were in stages I, II, III, and IV, respectively. Additionally, 80 patients had perineural invasion, 114 had vascular invasion, and 85 had poorly differentiated lesions.

The ACR values of all patients ranged from 0.021 to 157.826, with a mean value of 14.670 ± 15.559 and a median of 10.879. In this study, the median ACR was 6.24 (95% CI: 1.46–15.45) in patients with recurrent rectal cancer, while it was 12.43 (95% CI: 5.78–21.28) in those without recurrence (Supplementary Figure S1A). Similarly, the median ACR value for deceased patients was significantly lower than that of non-deceased patients [7.40 (95% CI: 1.84–15.28) vs. 12.96 (95% CI: 6.54–22.81)] (Supplementary Figure S1B). The ROC curve can determine the sensitivity and specificity of ACR as a predictor of survival in rectal cancer patients. The optimal cut-off value for ACR was 4.38, with an area under the curve (AUC) of 0.654 (*p* < 0.001), a sensitivity of 82.9%, and a specificity of 41.4% (Supplementary Figure S2).

A total of 199 rectal cancer patients were found to have a low ACR (<4.38), while 537 patients with rectal cancer had a high ACR (≥4.38). Low ACR was significantly associated with advanced age; T, N, and M stages; larger tumor diameter; low albumin levels; and high CEA levels. Patients in the low ACR group had a 22.7% higher recurrence and 29.8% higher mortality rates than those in the high ACR group. Additionally, patients in the low ACR group had longer hospital stays (2 days) and higher hospitalization costs (>2,000 RMB) than those in the high ACR group. These findings suggest that a low ACR is associated with worse pathological states, poorer outcomes, and higher medical costs for patients (Table 1).

**Table 1:** The relationships between the ACR and clinicopathological characteristics of patients with rectal cancer.

Clinicopathological characteristics	All patients (*n* = 736)	ACR		*p*-value
		Low (*n* = 199)	High (*n* = 537)	
Sex (Man)	461 (62.6)	133 (66.8)	328 (61.1)	0.178
Age [mean (SD)]	58.15 (12.87)	59.73 (12.57)	57.56 (12.95)	0.042
BMI (median [IQR])	22.06 (20.06, 24.42)	22.22 (20.20, 24.22)	21.97 (20.00, 24.45)	0.823
Hypertension (yes)	108 (14.7)	33 (16.6)	75 (14.0)	0.439
Diabetes (yes)	36 (4.9)	11 (5.5)	25 (4.7)	0.768
T stage (T3-4)	509 (69.2)	166 (83.4)	343 (63.9)	<0.001
N stage				<0.001
N0	415 (56.4)	88 (44.2)	327 (60.9)	
N1	186 (25.3)	56 (28.1)	130 (24.2)	
N2	135 (18.3)	55 (27.6)	80 (14.9)	
M stage	49 (6.7)	34 (17.1)	15 (2.8)	<0.001
TNM stage				<0.001
Stage I	171 (23.2)	23 (11.6)	148 (27.6)	
Stage II	231 (31.4)	54 (27.1)	177 (33.0)	
Stage III	285 (38.7)	88 (44.2)	197 (36.7)	
Stage IV	49 (6.7)	34 (17.1)	15 (2.8)	
Perineural invasion (yes)	80 (10.9)	28 (14.1)	52 (9.7)	0.118
Vascular invasion (yes)	114 (15.5)	37 (18.6)	77 (14.3)	0.193
Macroscopic type				0.078
Protrude type	183 (24.9)	38 (19.1)	145 (27.0)	
Infiltrating type	57 (7.7)	15 (7.5)	42 (7.8)	
Ulcerative type	496 (67.4)	146 (73.4)	350 (65.2)	
Differentiation (poor)	85 (11.5)	22 (11.1)	63 (11.7)	0.9
Tumor size (median [IQR])	4.00 (3.00, 5.00)	5.00 (4.00, 5.50)	4.00 (3.00, 5.00)	<0.001
CEA (High)	3.59 (2.05, 10.11)	22.93 (13.30, 45.12)	2.70 (1.65, 4.09)	<0.001
Album	39.40 (36.90, 41.60)	38.50 (36.10, 41.05)	39.70 (37.40, 41.70)	0.001
Radiotherapy (yes)	127 (17.3)	26 (13.1)	101 (18.8)	0.085
Chemotherapy (yes)	363 (49.3)	102 (51.3)	261 (48.6)	0.578
Death (yes)	302 (41.0)	125 (62.8)	177 (33.0)	<0.001
Recurrence (yes)	218 (29.6)	92 (46.2)	126 (23.5)	<0.001
Length of stay (median [IQR])	18.00 (11.00, 22.00)	19.00 (12.00, 23.00)	17.00 (11.00, 22.00)	0.042
Hospitalization cost (median [IQR])	50621.62 (45787.65, 57012.92)	52146.26 (46387.85, 61041.58)	49948.86 (45473.69, 55660.61)	0.004

BMI, body mass index; ACR, albumin-to-carcinoembryonic antigen ratio.

### Relationship between ACR and recurrence

**Figure 1:**
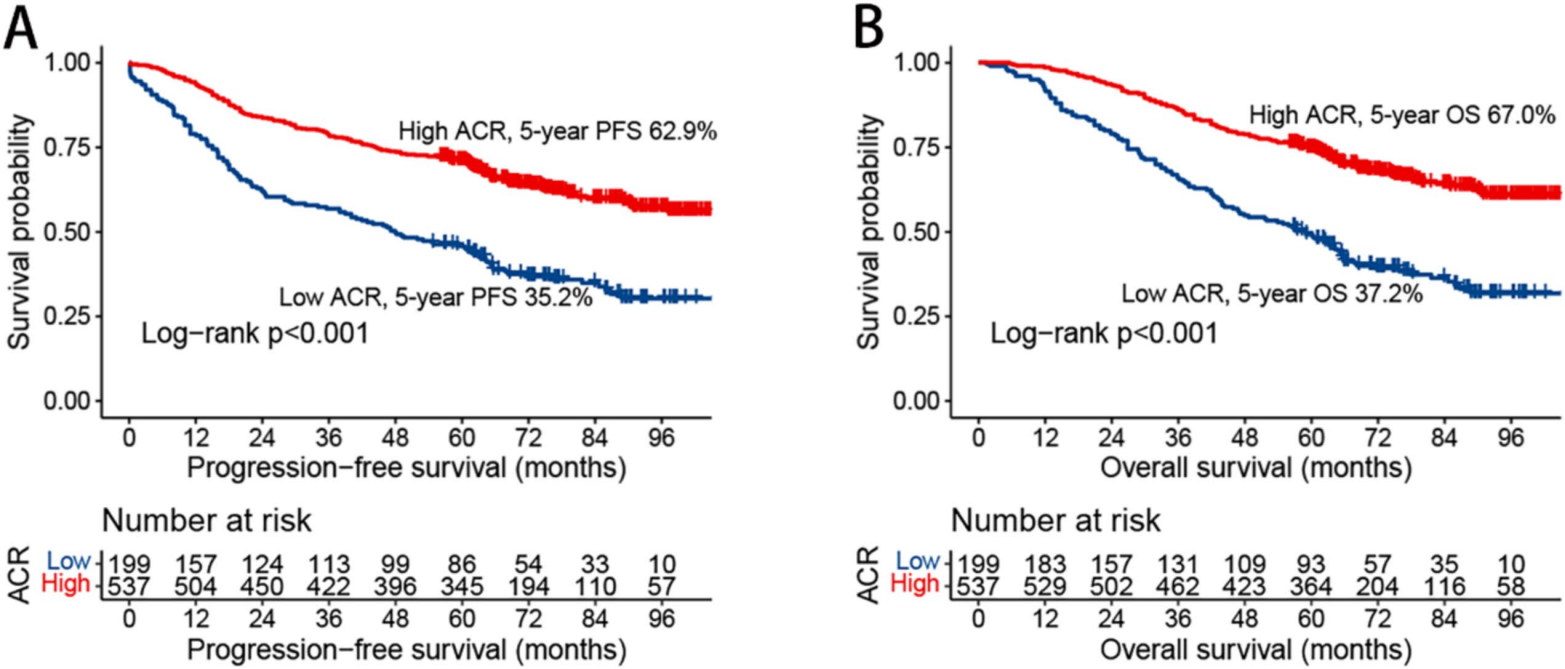
Kaplan–Meier curve of albumin-carcinoembryonic antigen ratio in patients with rectal cancer. **(A)** Progression-free survival; **(B)** overall survival; ACR, albumin-to-carcinoembryonic antigen ratio; PFS, progression-free survival; OS, overall survival.

**Figure 2:**
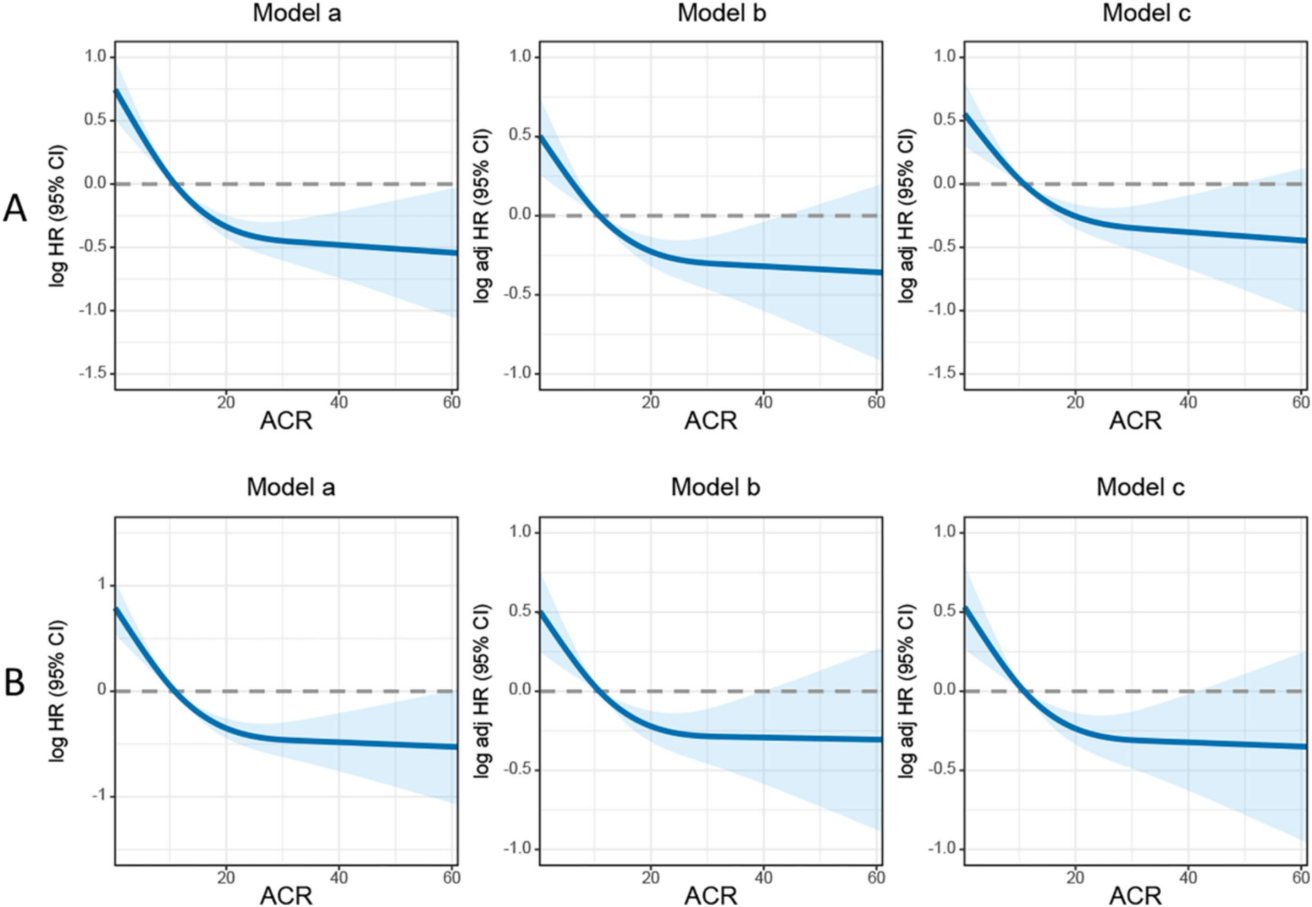
The association between albumin-carcinoembryonic antigen ratio and survival in patients with rectal cancer. **(A)** Progression-free survival; **(B)** overall survival. Model a: no adjusted. Model b: adjusted for gender, age, and BMI. Model c: adjusted for gender, age, BMI, hypertension, diabetes, T stage, N stage, tumor size, perineural invasion, vascular invasion, macroscopic type, differentiation, radiotherapy, chemotherapy. ACR, albumin-to-carcinoembryonic antigen ratio.

In this study, we observed that rectal cancer patients with a high ACR had a significantly higher 5-year PFS than those with a low ACR (62.9% vs. 35.2%, *p* < 0.001) (Figure 1A). Subgroup analysis revealed that ACR significantly stratified the prognosis of patients with stages III–IV rectal cancer (74.6% vs. 62.0%, *p* = 0.002), but not of those with stages I–II rectal cancer (Supplementary Figures S3A,B). We investigated the relationship between ACR as a continuous variable and recurrence outcomes in rectal cancer patients. We observed a significant L-shaped association between ACR and tumor recurrence, indicating that as ACR increased, the risk of tumor recurrence gradually decreased. This association remained significant even after adjusting for confounding factors (Figure 2A). In the univariate Cox regression analysis, ACR emerged as an important factor affecting the recurrence of rectal cancer (HR = 0.440, 95% CI: 0.352–0.549, *p* < 0.001). After adjusting for factors with *p* < 0.05 in the univariate analysis, multivariate Cox regression analysis revealed that ACR remained an independent factor affecting the recurrence of rectal cancer (HR = 0.581, 95% CI: 0.458–0.738, *p* < 0.001) (Table 2). Additionally, subgroup analysis of multivariate PFS indicated that in most subgroups, the HR of high ACR was distributed to the left of 1, indicating a good prognostic value of ACR in most subgroups. We also observed a significant interaction between ACR and N stage (*p* = 0.033). As the N stage progressed, the protective effect of high ACR became stronger, suggesting that ACR may be more suitable for the prognostic evaluation of patients with progressive N stage (Supplementary Figure S4A).

**Table 2:** Univariate and multivariate Cox regression analysis of clinicopathological characteristics associated with progression-free survival in patients with rectal cancer.

Clinicopathological characteristics	Progression-free survival			
	Univariate analysis		Multivariate analysis	
	HR (95%CI)	*P*-value	HR (95%CI)	*P*-value
Age	1.291 (1.038–1.604)	0.021	1.41 (1.126–1.765)	0.003
T stage (T3-4)	2.429 (1.835–3.214)	<0.001	1.54 (1.134–2.091)	0.006
N stage				
N0	Ref.			
N1	1.615 (1.243–2.099)	<0.001	1.381 (1.054–1.809)	0.019
N2	3.144 (2.419–4.086)	<0.001	2.237 (1.662–3.011)	<0.001
M stage	5.633 (4.1–7.74)	<0.001	3.075 (2.174–4.351)	<0.001
Perineural invasion (Positive)	1.873 (1.392–2.52)	<0.001	1.178 (0.851–1.631)	0.323
Vascular invasion (Positive)	1.98 (1.526–2.57)	<0.001	1.186 (0.875–1.608)	0.272
Pathological type				
Protrude type	Ref.			
Infiltrating type	1.499 (0.965–2.327)	0.072	1.008 (0.634–1.602)	0.973
Ulcerative type	1.491 (1.129–1.969)	0.005	1.18 (0.884–1.576)	0.261
Differentiation (High-medium)	0.672 (0.491–0.921)	0.013	0.729 (0.525–1.013)	0.060
ACR (High)	0.44 (0.352–0.549)	<0.001	0.581 (0.458–0.738)	<0.001

BMI, body mass index; ACR, albumin-to-carcinoembryonic antigen ratio.

### Relationship between ACR and survival

Kaplan–Meier analysis demonstrated that patients with a high ACR had a significantly higher OS than those with a low ACR (67.0% vs. 37.2%, *p* < 0.001) (Figure 1B). Subgroup analysis showed that ACR effectively differentiated the prognosis of patients with stages I–II and III–IV cancer. For stages I–II, patients in the high ACR group had a significantly longer OS than those in the low ACR group (74.2% vs. 59.7%, *p* = 0.020). For stages III–IV, ACR significantly stratified the prognosis of rectal cancer patients (56.1% vs. 23.0%, *p* < 0.001) (Supplementary Figures S3C,D). The association between ACR on a continuous scale and the risk of mortality was L-shaped (Figure 2B). In the univariate analysis, patients with a high ACR had a significantly better prognosis than those with a low ACR (HR = 0.410, 95% CI: 0.326–0.516, *p* < 0.001). Multivariate Cox regression analysis showed that ACR was an independent prognostic factor for predicting OS of rectal cancer patients (HR = 0.560, 95% CI: 0.435–0.720, *p* < 0.001) (Table 3). Multivariate subgroup analysis demonstrated that ACR was an effective indicator for predicting OS in most patient subgroups (Supplementary Figure S4B).

**Table 3:** Univariate and multivariate Cox regression analysis of clinicopathological characteristics associated with overall survival in patients with rectal cancer.

Clinicopathological characteristics	Overall survival			
	Univariate analysis		Multivariate analysis	
	HR (95%CI)	*P*-value	HR (95%CI)	*P*-value
Age	1.402 (1.117–1.761)	0.004	1.483 (1.172–1.877)	0.001
T stage (T3-4)	2.598 (1.922–3.511)	<0.001	1.648 (1.187–2.288)	0.003
N stage				
N0	Ref.		Ref.	
N1	1.61 (1.223–2.119)	0.001	1.374 (1.034–1.825)	0.028
N2	3.121 (2.38–4.092)	<0.001	2.068 (1.518–2.817)	<0.001
M stage	5.013 (3.637–6.911)	<0.001	2.656 (1.867–3.779)	<0.001
Perineural invasion (Positive)	1.762 (1.29–2.406)	<0.001	1.081 (0.766–1.526)	0.657
Vascular invasion (Positive)	2.024 (1.548–2.647)	<0.001	1.274 (0.93–1.746)	0.132
Pathological type				
Protrude type	Ref.		Ref.	
Infiltrating type	1.547 (0.977–2.449)	0.063	1.059 (0.654–1.716)	0.815
Ulcerative type	1.5 (1.119–2.013)	0.007	1.159 (0.854–1.571)	0.343
Differentiation (high-medium)	0.632 (0.459–0.871)	0.005	0.66 (0.47–0.926)	0.016
Tumor size	1.435 (1.141–1.805)	0.002	1.065 (0.837–1.355)	0.609
ACR (high)	0.41 (0.326–0.516)	<0.001	0.56 (0.435–0.72)	<0.001

BMI, body mass index; ACR, albumin-to-carcinoembryonic antigen ratio.

### Validation of the relationship between ACR and survival

To further validate the clinical efficacy of ACR in rectal cancer patients, we randomly assigned the entire population into two validation cohorts in a 7:3 ratio: A (516 cases) and B (220 cases). No statistically significant differences were observed in the clinicopathological characteristics between the two cohorts (Supplementary Table S1). In validation cohort A, patients in the high ACR group had significantly longer PFS (62.8% vs. 35.9%, *p* < 0.001) (Supplementary Figure S5A) and OS (66.3% vs. 38.7%, *p* < 0.001) (Supplementary Figure S5B) than those in the low ACR group. Multivariate Cox regression analysis revealed that ACR was an independent predictor of PFS (HR = 0.508, 95% CI: 0.381–0.678, *p* < 0.001) and OS (HR = 0.512, 95% CI: 0.380–0.690, *p* < 0.001) in rectal cancer patients. In validation cohort B, ACR effectively stratified the PFS (63.2% vs. 33.3%, *p* < 0.001) (Supplementary Figure S6A) and OS (68.7% vs. 33.3%, *p* < 0.001) (Supplementary Figure S6B) of patients. Multivariate Cox regression analysis showed that ACR remained a significant factor in evaluating PFS (HR = 0.579, 95% CI: 0.361–0.928, *p* = 0.023) and OS (HR = 0.496, 95% CI: 0.305–0.807, *p* < 0.001) in rectal cancer patients (Supplementary Table S2).

### Construction of ACR-based nomograms

**Figure 3:**
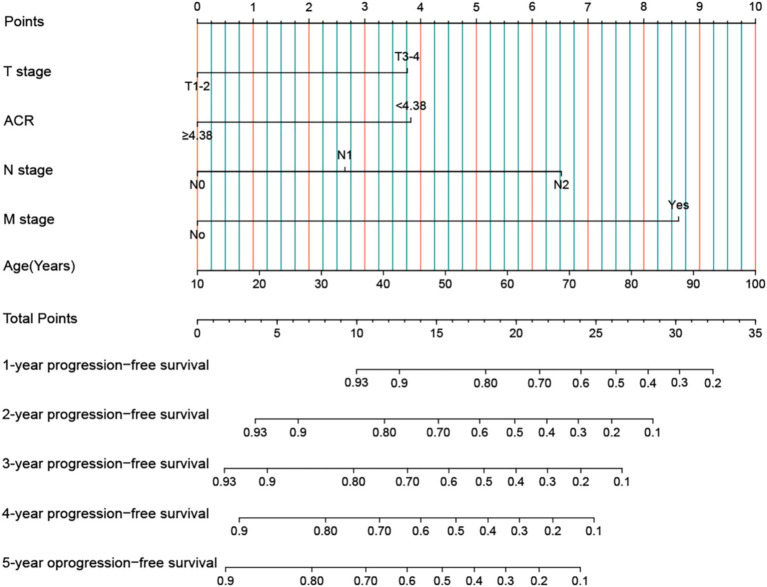
Construction the ACR-based PFS nomogram in patients with rectal cancer. ACR, albumin-to-carcinoembryonic antigen ratio; T stage, tumor stage; N stage, node stage; M stage, metastasis stage.

We developed an ACR-based PFS nomogram by incorporating independent prognostic factors identified through PFS in the multivariate Cox regression analysis, including T stage, ACR, N stage, M stage, and age (Figure 3). The nomogram showed that the predictive score increased with age, lower ACR, and more advanced pathological stage, indicating a greater likelihood of PFS. The C-index and calibration curve were used to evaluate the prognostic predictive ability of the nomograms. The nomogram’s C-index was 0.694 (95% CI: 0.665–0.723), and the calibration curve showed good consistency between the predicted and observed values at the 3- and 5-year points (Supplementary Figure S7).

**Figure 4:**
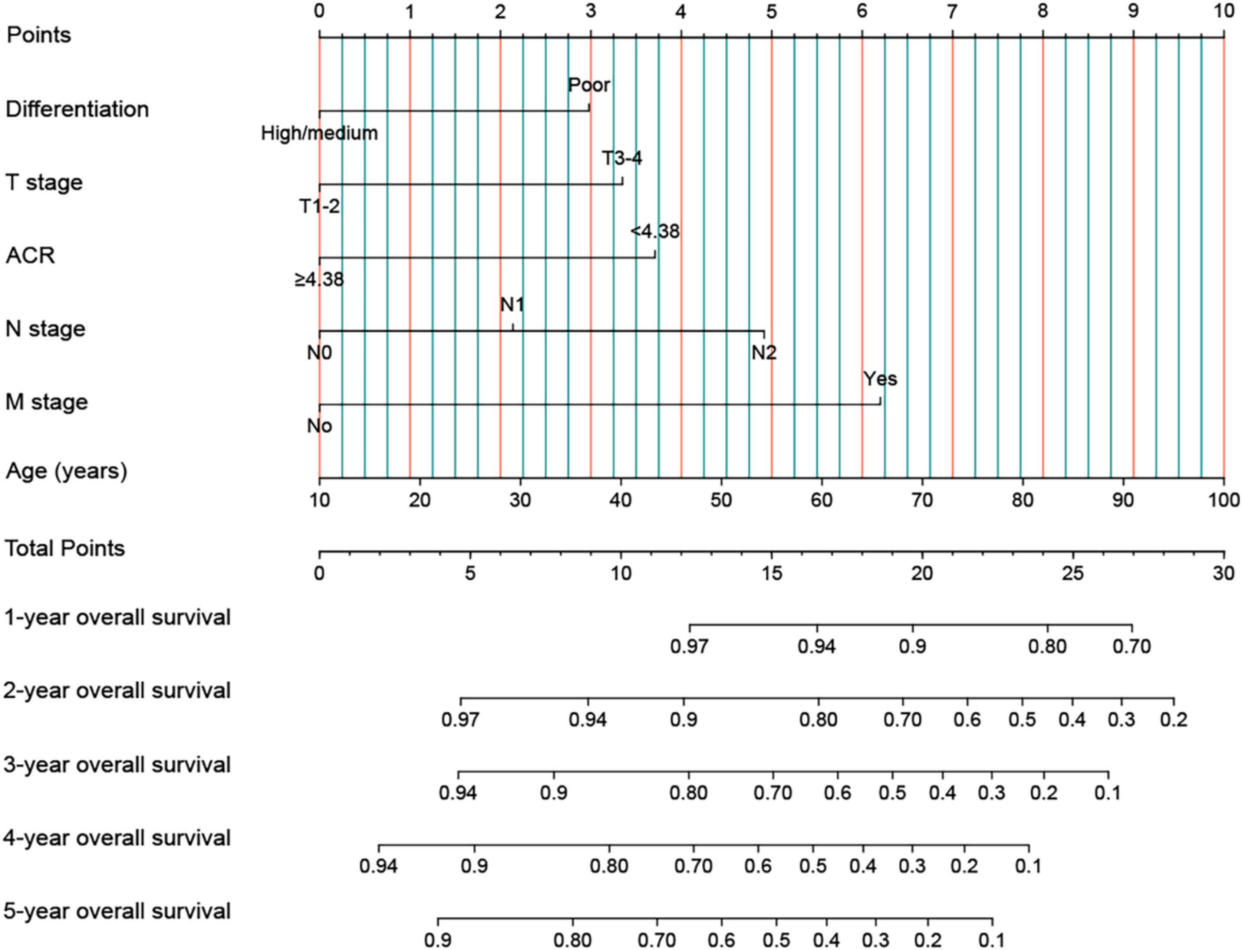
Construction the ACR-based OS nomograms in patients with rectal cancer. ACR, albumin-to-carcinoembryonic antigen ratio; T stage, tumor stage; N stage, node stage; M stage, metastasis stage.

In addition, we used indicators with *p* < 0.05 from OS in the multivariable Cox analysis to construct an OS nomogram for predicting the 1–5-year OS in rectal cancer patients (Figure 4). The OS nomogram included tumor differentiation, T stage, ACR, N stage, M stage, and age. As the total score of the nomogram increased, the clinical prognosis worsened. The OS nomogram’s C-index was 0.699 (95% CI: 0.668–0.730), and the calibration curve demonstrated good consistency between the actual observed survival rate and the survival rate predicted by the OS nomogram (Supplementary Figure S8).

### Comparison of ACR-based nomograms and traditional TNM stage

We compared the predictive ability of these nomograms with that of the traditional TNM stage system to predict the prognosis of rectal cancer patients. In terms of PFS, the ROC curves showed that the AUCs of the PFS nomogram were 3 and 4.3% higher than those of the traditional TNM staging system at the 3- and 5-year time points, respectively (Supplementary Figure S9). In terms of OS, the ROC curves showed that the AUC of the PFS nomogram was higher than that of the traditional TNM stage system at the 3- and 5-year time points (Supplementary Figure S10). The results of the C-index, cNRI, and IDI indicated that the PFS nomogram had improved prognostic predictive accuracy compared to the traditional TNM stage system, with increases of 4.5, 19.7, and 6.6%, respectively (all *p* < 0.001) (Table 4).

**Table 4:** Comparative analysis of the discrimination of the nomograms and TNM stage for mortality in training cohort.

Discrimination Ability	C-statistic			cNRI		IDI	
	Value	Difference	*p*-value	Difference	*p*-value	Difference	*p*-value
PFS							
TNM stage	0.659 (0.630, 0.687)	Ref		Ref		Ref	
PFS nomogram	0.693 (0.663,0.723)	0.035 (0.017, 0.052)	<0.001	0.155 (0.028,0.261)	0.014	0.055 (0.027,0.091)	<0.001
OS							
TNM stage	0.654 (0.623, 0.684)	Ref		Ref		Ref	
OS nomogram	0.699 (0.668, 0.731)	0.045 (0.024, 0.066)	<0.001	0.197 (0.091,0.312)	<0.001	0.066 (0.037,0.107)	<0.001

## Discussion

This study revealed that ACR is a powerful biomarker for predicting the recurrence and survival of rectal cancer patients. Low ACR levels are associated with a poor prognosis and an increased risk of disease recurrence. ACR reflects a patient’s nutritional status and anti-cancer abilities, and a low ACR may indicate malnutrition or advanced cancer. Notably, ACR levels were significantly increased in patients who had a recurrence or poor prognosis, and a low ACR was significantly correlated to stronger tumor invasiveness, higher hospitalization costs, and longer hospital stays. These findings suggest that a low ACR represents worse pathological states, outcomes, and higher medical costs for patients.

To the best of our knowledge, this study is the first to demonstrate the prognostic value of ACR in rectal cancer patients. ACR is significantly and inversely associated with the survival of rectal cancer patients in an L-shaped manner. As ACR increases, the risk of death from rectal cancer gradually decreases. We identified the optimal cut-off value of ACR in rectal cancer patients as 4.38, which effectively stratified the prognosis of rectal cancer patients. Furthermore, we conducted internal cohort validation to further validate the prognostic efficacy of ACR in rectal cancer, demonstrating that ACR has broad application prospects for predicting the prognosis of rectal cancer patients.

Although TNM stage is the most commonly used tool for prognostic prediction, efficacy evaluation, and treatment plan formulation in patients with rectal cancer, preoperative TNM stage is not yet available. Additionally, even patients in the same pathological stage have large variations in prognosis. This study found that ACR can serve as an effective auxiliary tool for pathological staging, further distinguishing the prognosis of rectal cancer patients with the same pathological stage, especially those with stages III–IV cancer. As a simple, economical, effective, and clinically friendly indicator, ACR has broad application prospects in the prognostic evaluation of rectal cancer patients.

The ACR combines the benefits of albumin and CEA to provide a more effective reflection of the host’s nutritional status, inflammatory status, and tumor burden. CEA, a traditional tumor marker, is crucial for evaluating disease burden, monitoring post-treatment progress, and determining prognosis because it is typically secreted by the tumor itself (15, 18). Serum CEA levels are widely used for the diagnosis, monitoring, and prediction of rectal cancer. A continuous increase in CEA levels before or after treatment may indicate tumor recurrence or metastasis. Egenvall et al. (19) found that an elevated CEA levels (>5 ng/mL) before or after treatment could predict an increased risk of recurrence and poor prognosis. Takagawa et al. (20) also found that preoperative serum CEA levels were predictive factors for the postoperative recurrence of CRC. Although the specificity of serum CEA as a method for detecting rectal cancer recurrence is high, its sensitivity is low, limiting its usefulness (21). Previous studies have shown that albumin is associated with the recurrence, metastasis, and poor prognosis of CRC patients. Wei et al. (22) found that albumin levels were significantly reduced in patients with metastatic CRC and were an independent prognostic factor for PFS in patients with metastatic CRC. González-Trejo et al. (23) believe that baseline serum albumin level is an important and independent prognostic factor for patients with CRC, and its influence remains unchanged among TNM stage and other known clinical prognostic factors. A low ACR suggests a poor prognosis and may indicate higher CEA and lower albumin levels in patients. This may reflect a higher degree of tumor invasiveness and later staging, as well as poor host nutrition and inflammatory status.

Diagnosis or treatment decisions should not solely rely on ACR. Typically, ACR must be used in conjunction with other tests and clinical information for a more comprehensive prognostic assessment. Nomograms are considered simple and effective tools for providing personalized risk prediction for patients (24). They can integrate many factors to predict the risk of specific events. In this study, we established ACR-based nomograms to predict the prognosis of rectal cancer patients based on independent factors affecting prognosis as determined by multivariate survival analysis. These nomograms can integrate personal conditions, tumor characteristics, and nutrition- and inflammation-related biomarkers, providing a more comprehensive prognostic assessment for rectal cancer patients. Compared to traditional TNM stage, these nomograms have a higher accuracy in predicting the prognosis of rectal cancer patients. These models can directly help to quantify the prognostic risk of rectal cancer patients, making it easier to formulate appropriate treatment strategies for these patients.

This study has several limitations. First, it was designed retrospectively and conducted at a single institution, which may have introduced selection bias. Therefore, external validation using a larger multicenter sample is necessary to confirm these findings. Second, the nomogram based on the ACR was developed using retrospectively collected data from a single-center patient cohort. Further research is required to validate its performance with larger external validation cohorts. Finally, the cutoff value for ACR was derived from a single study population. This cutoff requires validation across multiple cohorts before it can be widely applied in diverse populations.

## Conclusion

A decrease in ACR reflects more aggressive biological behavior, severe inflammation, and malnutrition in patients with rectal cancer, indicating a poor prognosis. The ACR is a simple and effective clinical tool for predicting the recurrence and survival of patients with rectal cancer. The ACR-based nomogram has a good predictive accuracy and can help optimize the prognosis, efficacy, and treatment for patients with rectal cancer.

## Data Availability

The raw data supporting the conclusions of this article will be made available by the authors, without undue reservation.
