# Supplementary material for: Serum albumin-carcinoembryonic antigen ratio as an effective clinical tool for predicting recurrence and overall survival in patients with rectal cancer
Source: Front Nutr. 2025 Jan 17;11:1521691. doi: 10.3389/fnut.2024.1521691 (PMC11782030; doi:10.3389/fnut.2024.1521691)
Supplement: Supplementary file 1 [file Supplementary_file_1.docx]

**Figure S1.** Median ACR of patients alive and with recurrence or death during follow-up

**
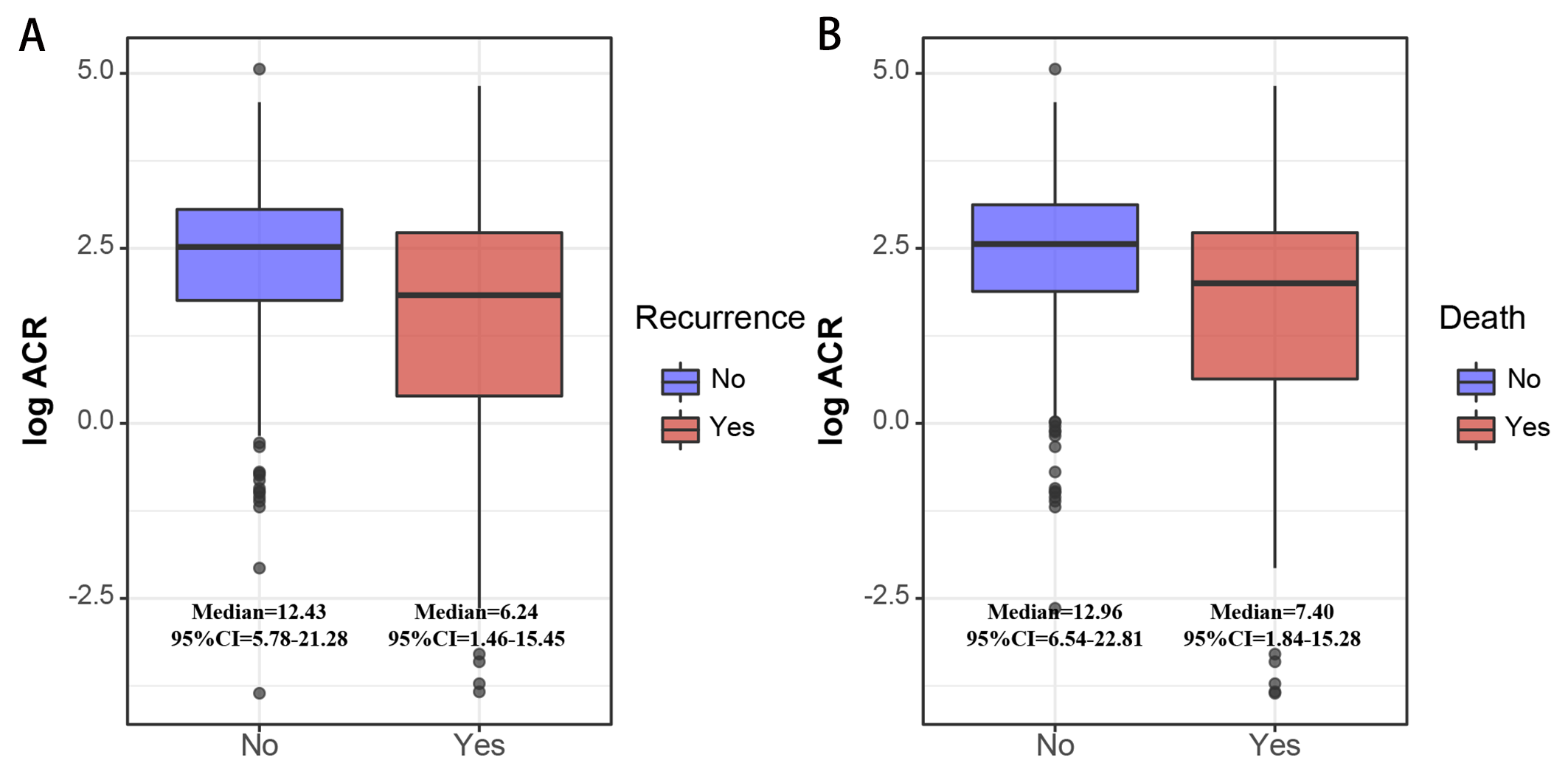
**

**Notes:** ACR, albumin-to-carcinoembryonic antigen ratio.

**Figure S2.** The optimal threshold of ACR in patients with rectal cancer.

**
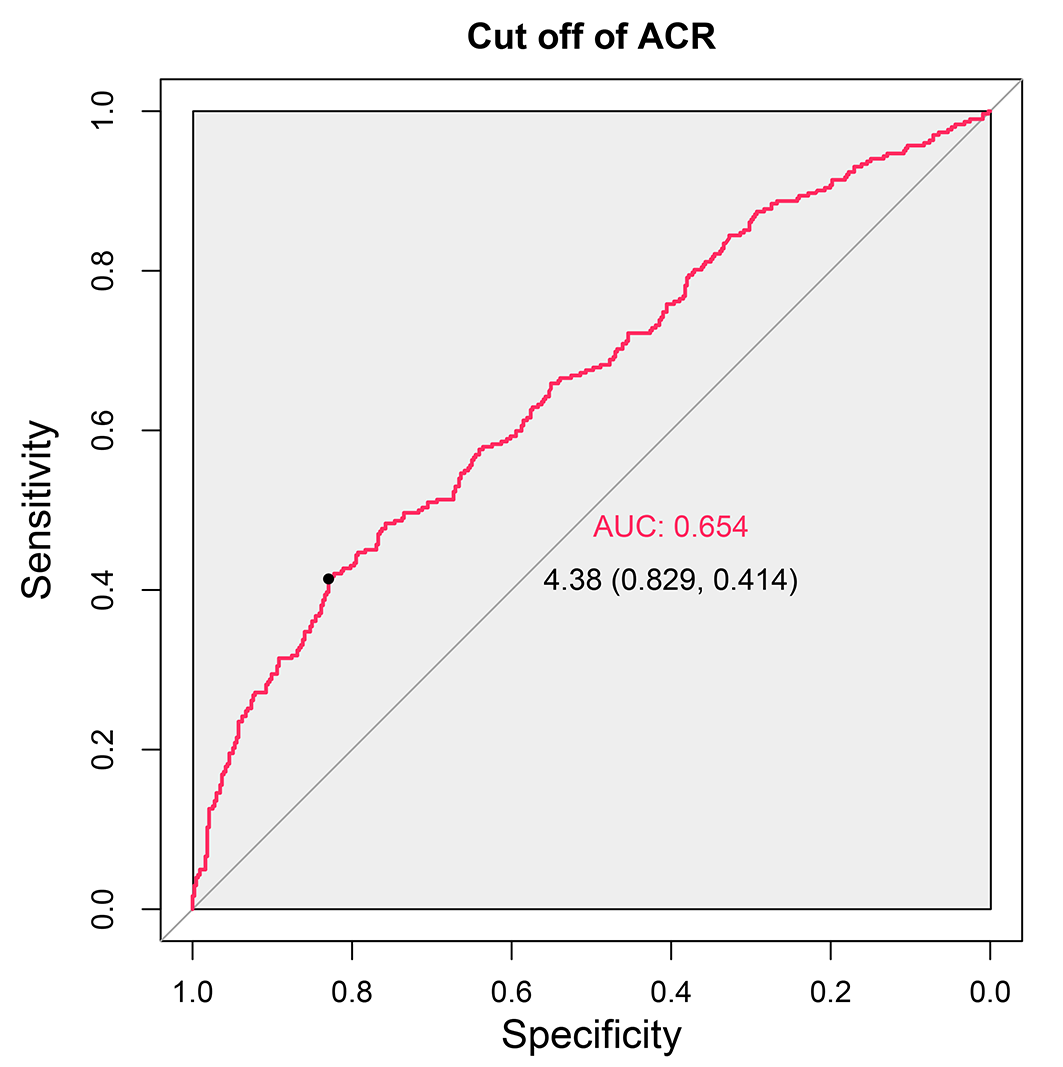
**

**Notes:** ACR, albumin-to-carcinoembryonic antigen ratio.

**Figure S3.** Stratified Kaplan-Meier curve of ACR based on TNM stage in patients with rectal cancer.


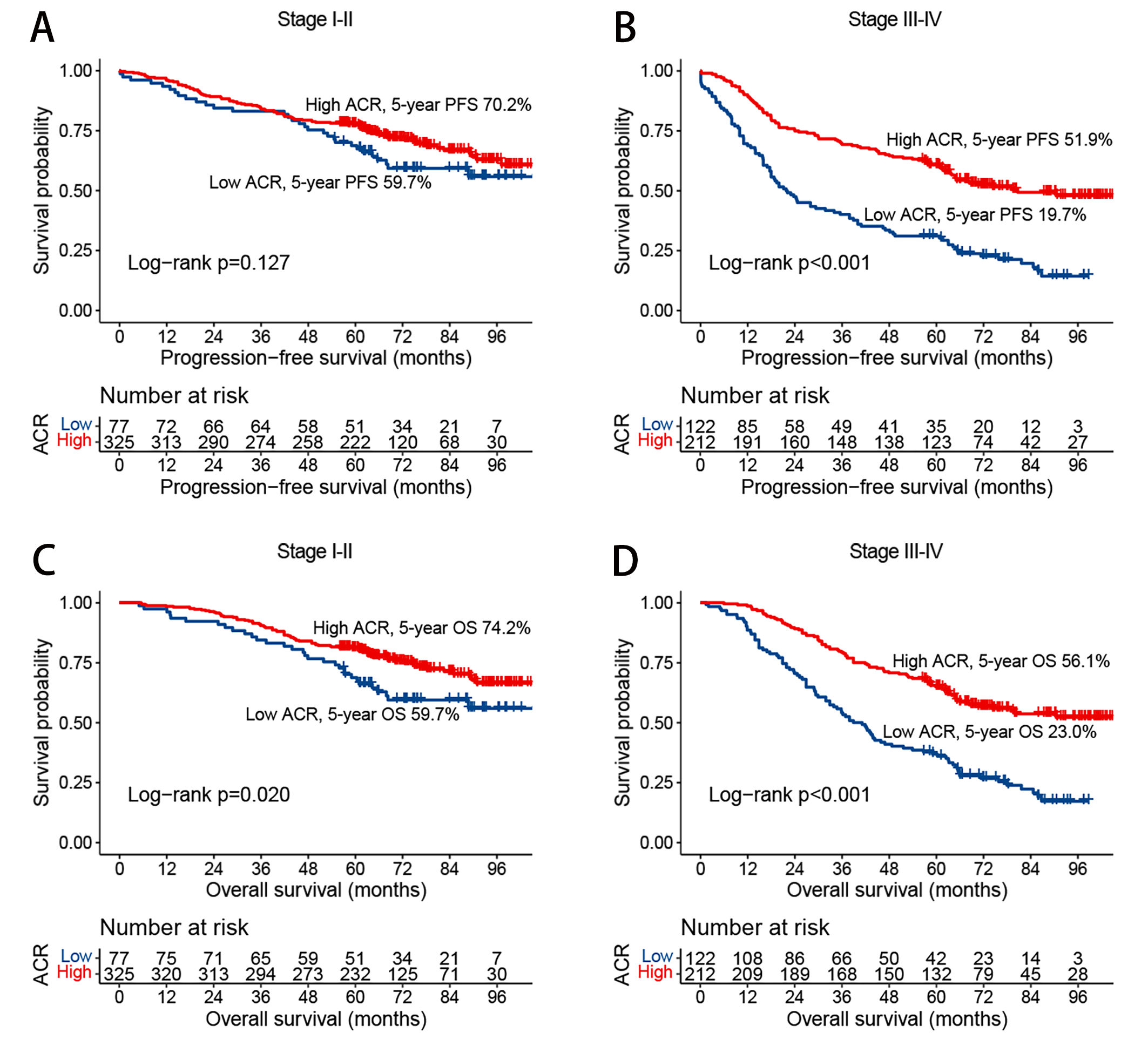


**Notes:** A, Progression-free survival of TNM I-II stage; B, Progression-free survival of TNM III-IV stage; C, Overall survival of TNM I-II stage; D, Overall survival of TNM III-IV stage; ACR, albumin-to-carcinoembryonic antigen ratio; TNM stage, Tumor Node Metastasis stage.

**Figure S4.** The association between ACR and hazard ratio of PFS and OS in various subgroups.


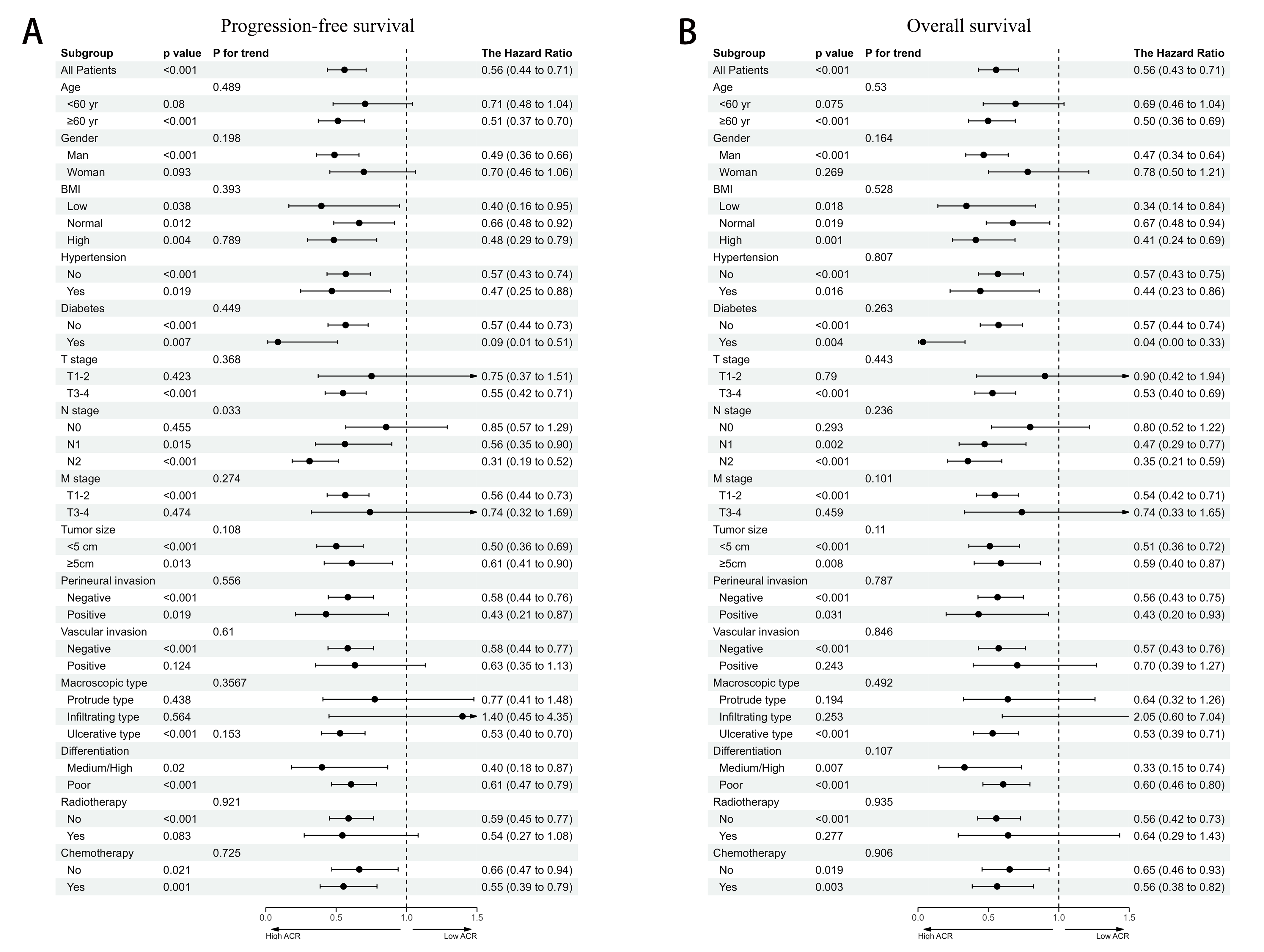


**Notes:** A, Progression-free survival, B, Overall survival; BMI, body mass index; ACR, albumin-to-carcinoembryonic antigen ratio; TNM stage, Tumor Node Metastasis stage; ACR, albumin-to-carcinoembryonic antigen ratio.

**Figure S5.** Kaplan-Meier curve of ACR in patients with rectal cancer at internal validation cohort A.

**
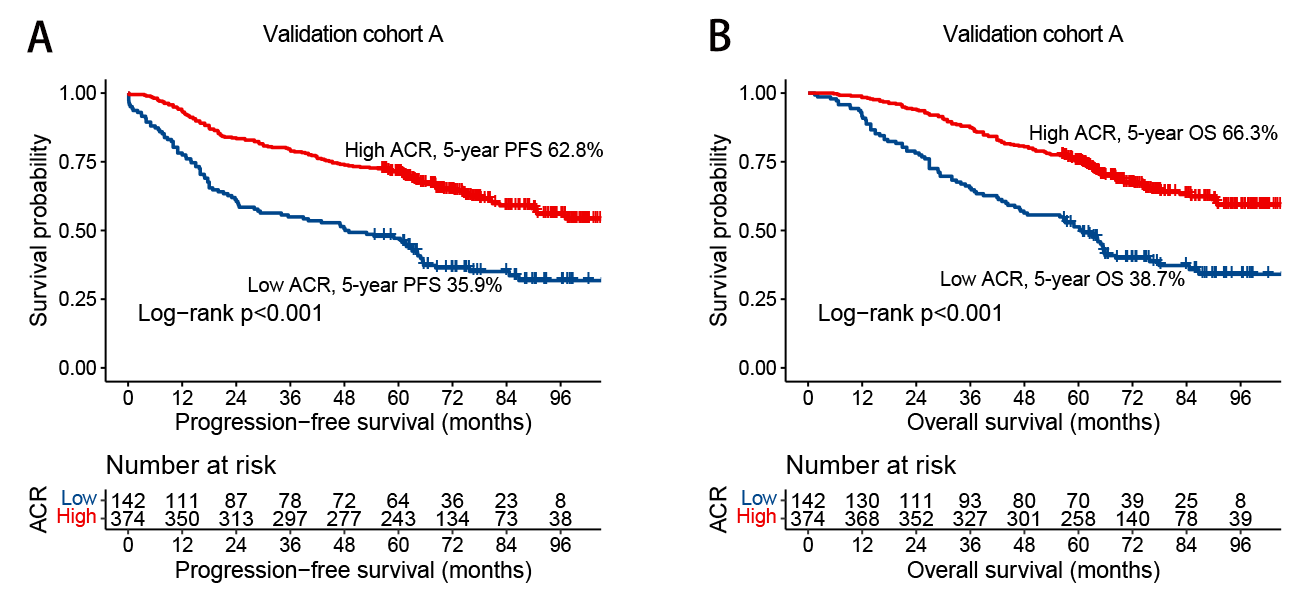
**

**Notes:** A, Progression-free survival at validation cohort A; B, Overall survival at validation cohort A; ACR, albumin-to-carcinoembryonic antigen ratio.

**Figure S6.** Kaplan-Meier curve of ACR in patients with rectal cancer at internal validation cohort B.

**
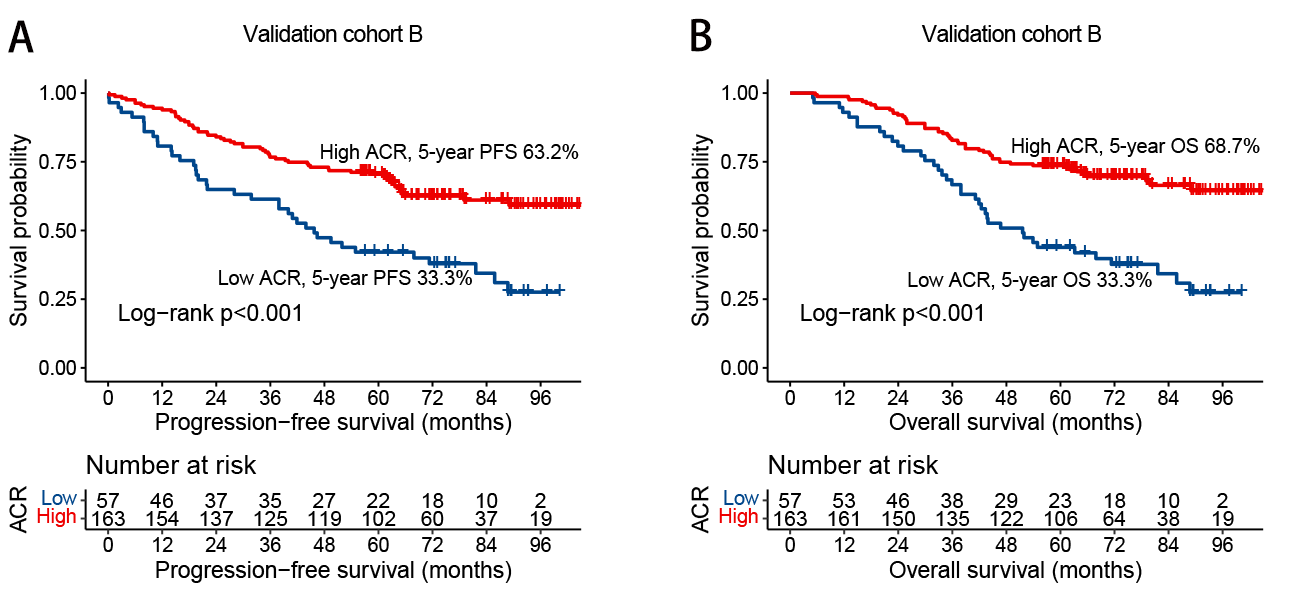
**

**Notes:** A, Progression-free survival at validation cohort B; B, Overall survival at validation cohort B. ACR, albumin-to-carcinoembryonic antigen ratio.

**Figure S7.** The calibration curve of the PFS nomograms in patients with rectal cancer.

**
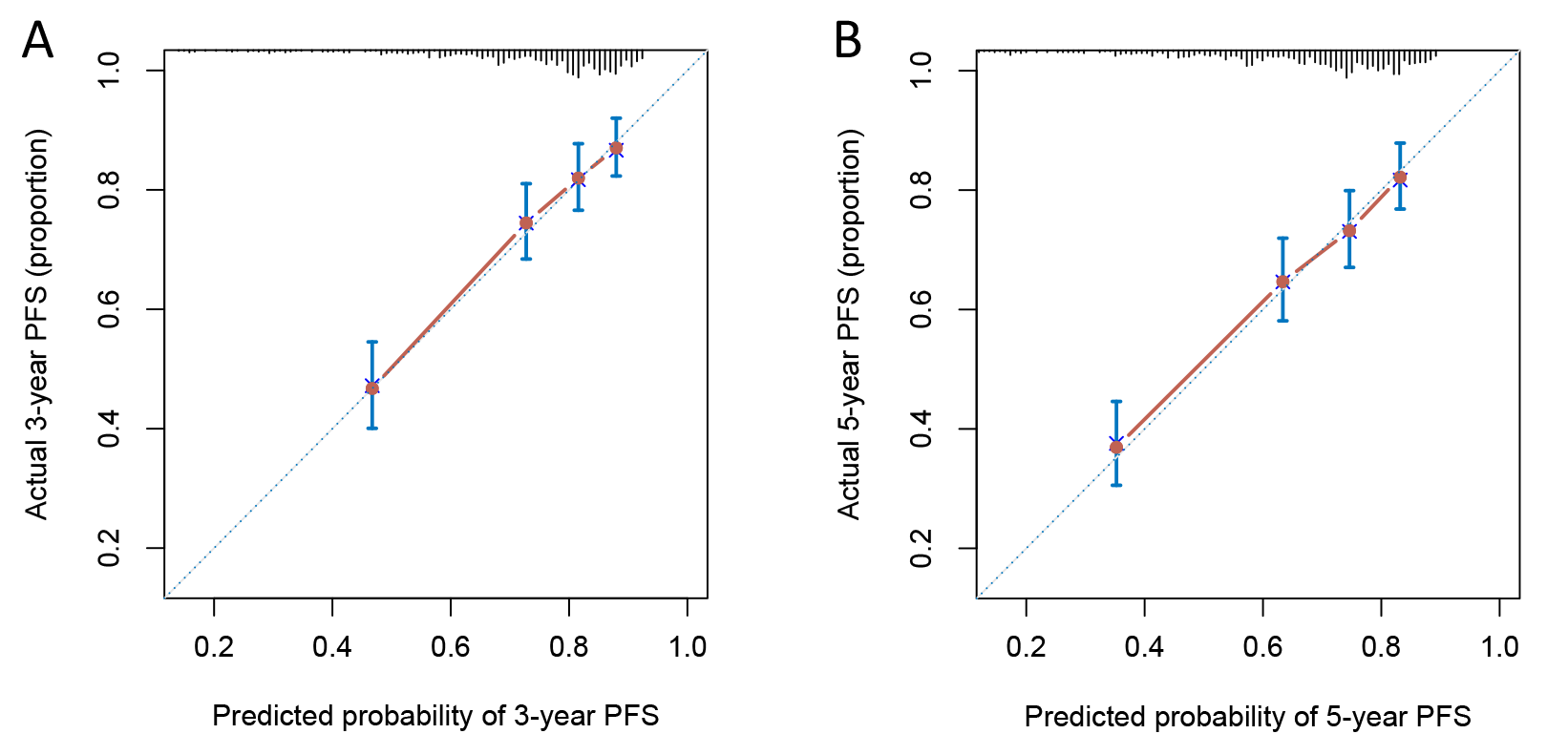
**

**Notes:** A, 3-year PFS; B, 5-year PFS; PFS, Progression-free survival.

**Figure S8.** The calibration curve of the OS nomograms in patients with rectal cancer.

**
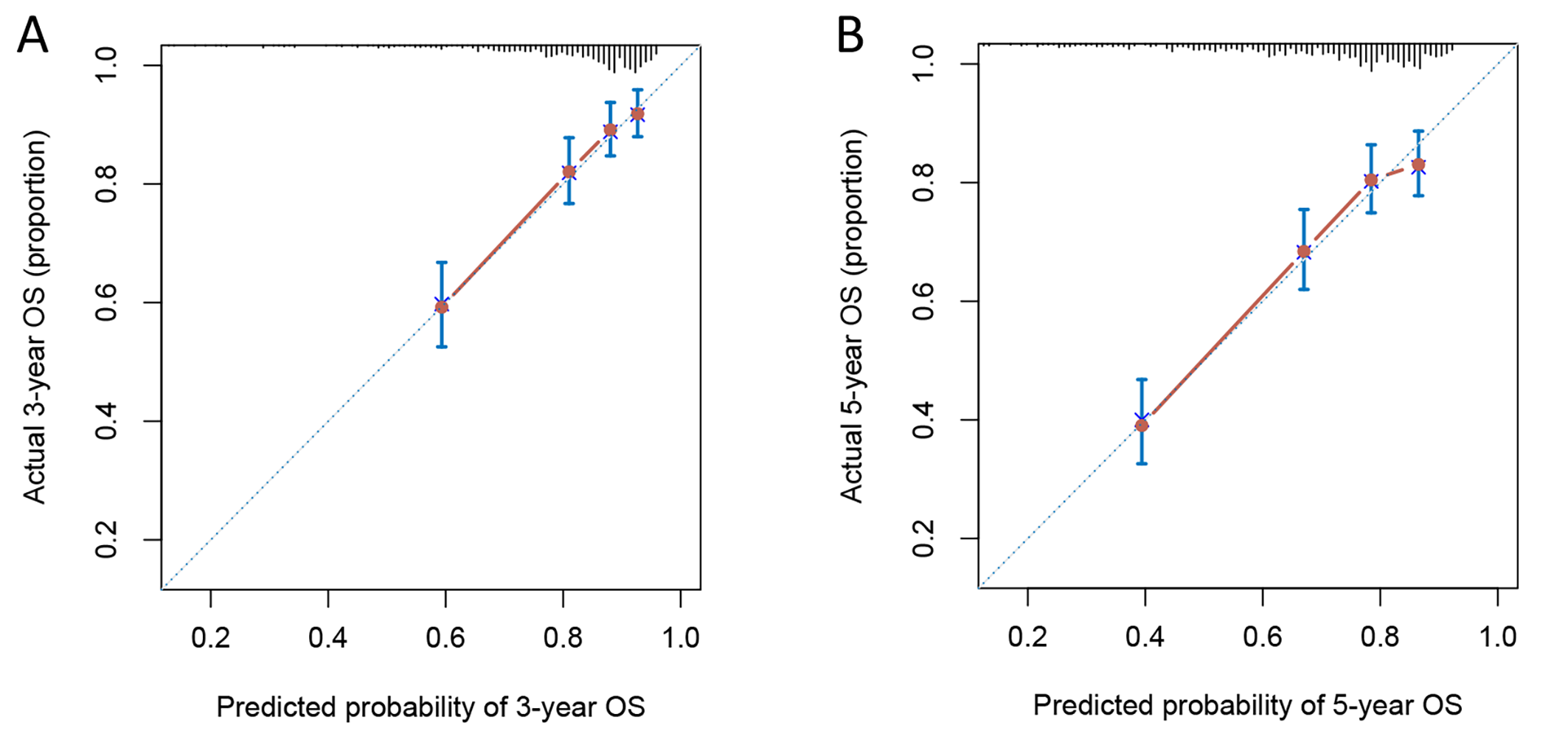
**

**Notes:** A, 3-year OS; B, 5-year OS; OS, Overall survival.

**Figure S9.** Comparison of the ability of the novel prognostic nomograms and TNM stage in predicting PFS of patients with rectal cancer.

**
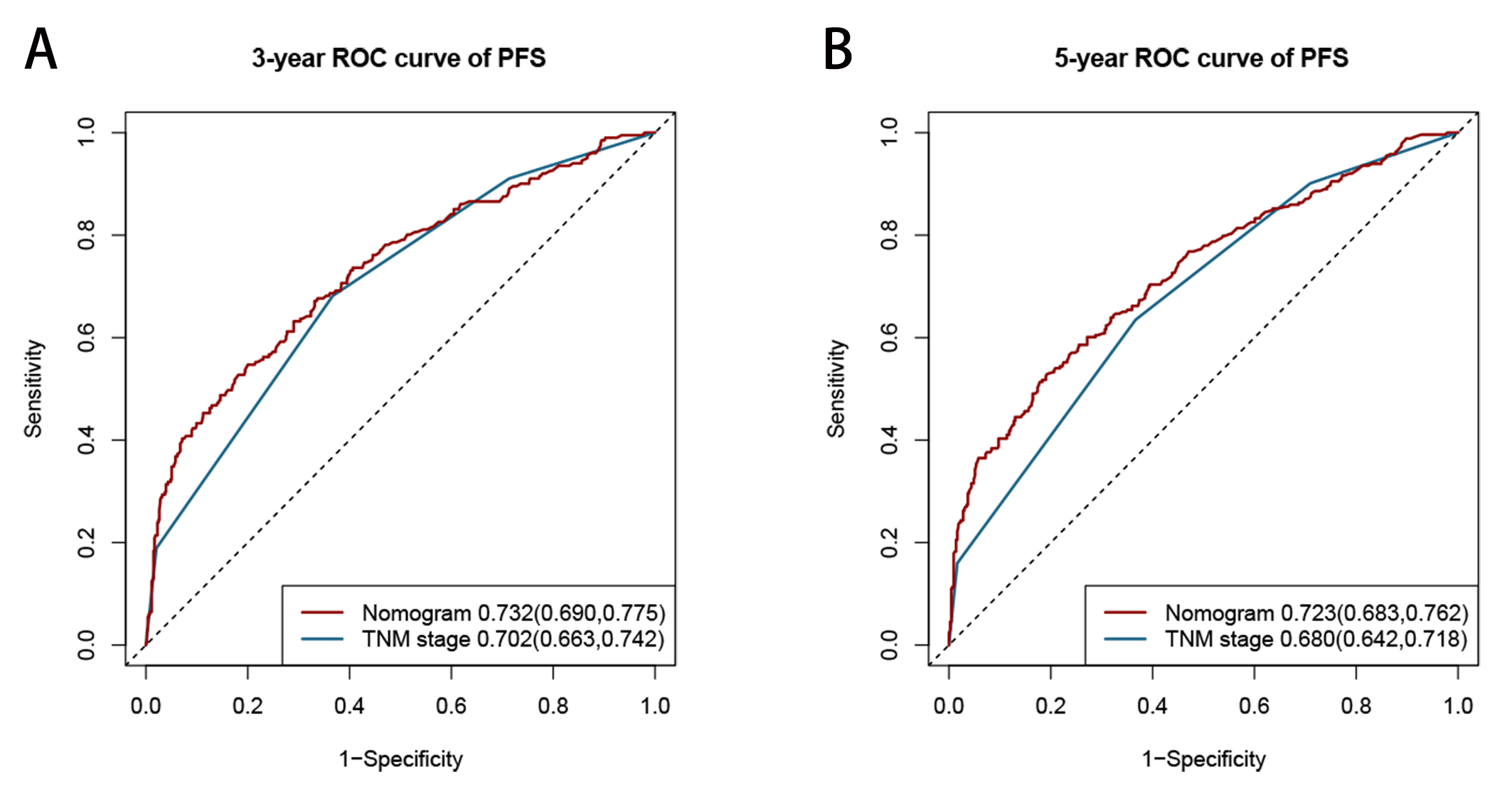
**

Notes: A, 3-year PFS; B, 5-year PFS; PFS, Progression-free survival; TNM stage, Tumor Node Metastasis stage.

**Figure S10.** Comparison of the ability of the novel prognostic nomograms and TNM stage in predicting OS of patients with rectal cancer.

**
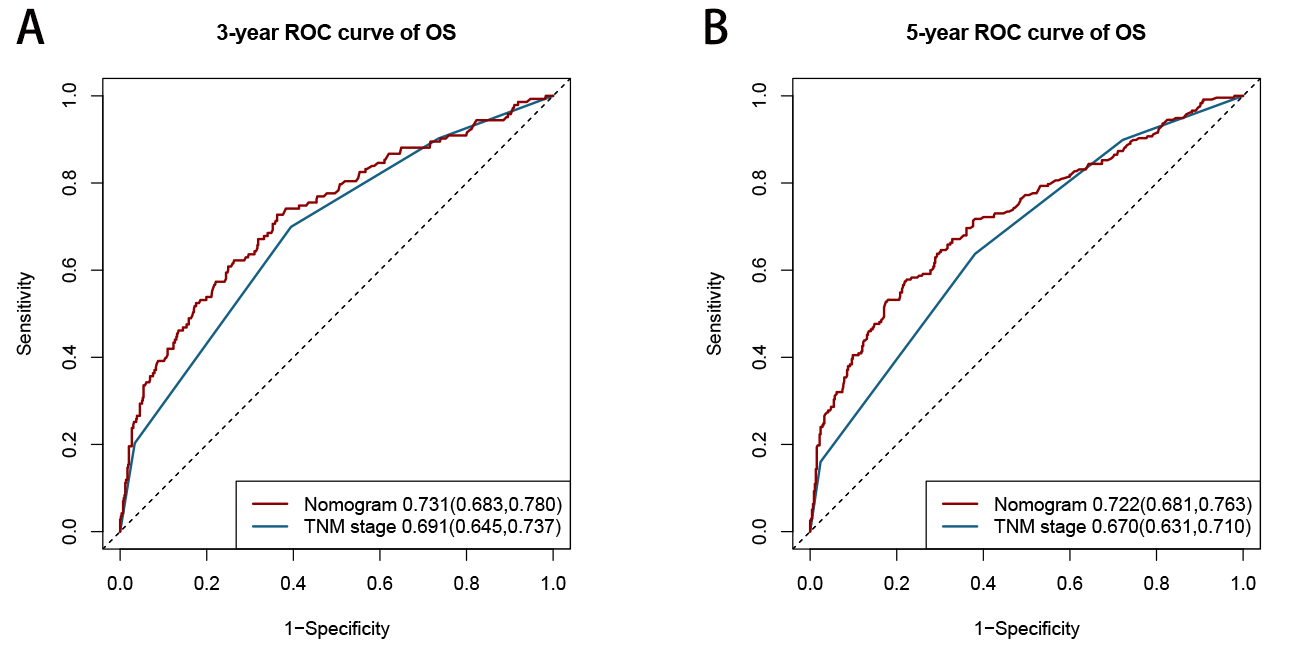
**

Notes: A, 3-year OS; B, 5-year OS; OS, Overall survival; TNM stage, Tumor Node Metastasis stage.

**Table S1.** The clinicopathological characteristics of two validation cohorts in patients with rectal cancer.

| Clinicopathological characteristics | Validation cohort A  (n = 516) | Validation cohort B  (n = 220) | p value |
| --- | --- | --- | --- |
| Sex (Man) | 327 (63.4) | 134 (60.9) | 0.583 |
| Age (mean (SD)) | 58.78 (12.90) | 56.65 (12.72) | 0.04 |
| BMI (median [IQR]) | 22.00 (20.10, 24.22) | 22.27 (19.92, 25.16) | 0.378 |
| Hypertension (Yes) | 74 (14.3) | 34 (15.5) | 0.782 |
| Diabetes (Yes) | 26 (5.0) | 10 (4.5) | 0.922 |
| T stage (T3-4) | 361 (70.0) | 148 (67.3) | 0.525 |
| N stage |  |  | 0.689 |
| N0 | 286 (55.4) | 129 (58.6) |  |
| N1 | 132 (25.6) | 54 (24.5) |  |
| N2 | 98 (19.0) | 37 (16.8) |  |
| TNM stage (III-IV) |  |  | 0.796 |
| Stage I | 119 (23.1) | 52 (23.6) |  |
| Stage II | 157 (30.4) | 74 (33.6) |  |
| Stage III | 205 (39.7) | 80 (36.4) |  |
| Stage IV | 35 (6.8) | 14 (6.4) |  |
| Perineural invasion (Yes) | 56 (10.9) | 24 (10.9) | 0.999 |
| Vascular invasion (Yes) | 79 (15.3) | 35 (15.9) | 0.925 |
| Macroscopic type |  |  | 0.498 |
| Protrude type | 125 (24.2) | 58 (26.4) |  |
| Infiltrating type | 37 (7.2) | 20 (9.1) |  |
| Ulcerative type | 354 (68.6) | 142 (64.5) |  |
| Differentiation (Poor) | 62 (12.0) | 23 (10.5) | 0.631 |
| Tumor size (median [IQR]) | 4.00 (3.00, 5.00) | 4.00 (3.00, 5.00) | 0.884 |
| CEA (High) | 3.82 (2.10, 10.37) | 3.10 (2.00, 9.66) | 0.093 |
| Albumin | 39.40 (37.10, 41.52) | 39.40 (36.68, 41.60) | 0.708 |
| Radiotherapy (Yes) | 83 (16.1) | 44 (20.0) | 0.238 |
| Chemotherapy (Yes) | 242 (46.9) | 121 (55.0) | 0.053 |
| Death (Yes) | 213 (41.3) | 89 (40.5) | 0.899 |
| Recurrence | 155 (30.0) | 63 (28.6) | 0.769 |
| Length of stay (median [IQR]) | 18.00 (11.00, 22.00) | 17.50 (11.00, 21.25) | 0.665 |
| Hospitalization cost (median [IQR]) | 51209.82 (46177.34, 57408.02) | 49861.05 (45002.07, 56702.47) | 0.144 |

**Table Notes:** BMI, body mass index; ACR, albumin-to-carcinoembryonic antigen ratio; TNM stage, Tumor Node Metastasis stage; SD, standard deviation; IQR, interquartile range; CEA, carcinoembryonic antigen.

**Table S2** Univariate and multivariate Cox regression analysis of clinicopathological characteristics associated with PFS and OS survival in patients with rectal cancer at validation cohorts.

| Clinicopathological characteristics | Univariate analysis | | Multivariate analysis | |
| --- | --- | --- | --- | --- |
|  | HR (95%CI) | p value | HR (95%CI) | p value |
| Validaiton cohort A | | | | |
| ACR (Low)^PFS^ | 0.441 (0.339-0.575) | <0.001 | 0.508 (0.381 - 0.678) | <0.001 |
| ACR (Low)^OS^ | 0.427 (0.324-0.561) | <0.001 | 0.512 (0.380 - 0.690) | <0.001 |
| Validaiton cohort B | | | | |
| ACR (Low)^PFS^ | 0.440 (0.293-0.662) | <0.001 | 0.579 (0.361 - 0.928) | 0.023 |
| ACR (Low)^OS^ | 0.378 (0.248-0.575) | <0.001 | 0.496 (0.305 - 0.807) | 0.005 |

**Table Notes:** Adjusted for gender, age, BMI, hypertension, diabetes, T stage, N stage, tumor size, perineural invasion, vascular invasion, macroscopic type, differentiation, radiotherapy, chemotherapy; PFS, progression-free survival; OS, overall survival; HR, hazard ratio; CI, confidence interval.
